# Diagnosis and Pharmacologic Management of Fibrotic Interstitial Lung Disease

**DOI:** 10.3390/life13030599

**Published:** 2023-02-21

**Authors:** Kristin Berger, Robert J. Kaner

**Affiliations:** 1 Division of Pulmonary and Critical Care Medicine, Department of Medicine, Weill Cornell Medicine, New York, NY 10021, USA; 2 Department of Genetic Medicine, Weill Cornell Medicine, New York, NY 10021, USA

**Keywords:** pulmonary fibrosis, interstitial lung disease, drug therapy, diagnostic techniques, procedures

## Abstract

Interstitial lung disease is an umbrella term that encompasses a spectrum of parenchymal lung pathologies affecting the gas exchanging part of the lung. While many of these disease entities are not fibrotic in nature, a number can lead to pulmonary fibrosis which may or may not progress over time. Idiopathic pulmonary fibrosis is the prototypical, progressive fibrotic interstitial lung disease, which can lead to worsening hypoxemic respiratory failure and mortality within a number of years from the time of diagnosis. The importance of an accurate and timely diagnosis of interstitial lung diseases, which is needed to inform prognosis and guide clinical management, cannot be overemphasized. Developing a consensus diagnosis requires the incorporation of a variety of factors by a multidisciplinary team, which then may or may not determine a need for tissue sampling. Clinical management can be challenging given the heterogeneity of disease behavior and the paucity of controlled trials to guide decision making. This review addresses current paradigms and recent updates in the diagnosis and pharmacologic management of these fibrotic interstitial lung diseases.

## 1. Introduction

Interstitial lung disease (ILD) includes a heterogeneous group of parenchymal lung diseases of various etiologies with a wide range of clinical manifestations and outcomes. While over 100 distinct ILDs have been identified, a subset of these disorders is most commonly encountered clinically, including idiopathic pulmonary fibrosis (IPF), sarcoidosis, hypersensitivity pneumonitis (HP), and connective tissue disease-associated ILDs (CTD-ILDs) [1]. A subset of ILDs, including those aforementioned, can result in pulmonary fibrosis, with the risk of progression and prognosis dependent upon the underlying disease process.

Diagnosing a patient with a specific ILD oftentimes proves challenging even for expert clinicians, particularly as definitions and diagnostic criteria for various ILDs have undergone revisions over the years [2]. Despite this difficulty, the early recognition and accurate diagnosis of ILD have major implications for a patient’s prognosis and management plan.

In this article, we review current approaches, with an emphasis on recent developments, in the diagnosis and pharmacologic management of fibrotic ILDs.

## 2. Diagnostic Approach

ILDs should be considered in the differential diagnosis of adults presenting with unexplained exertional shortness of breath, chronic cough, and/or crackles on chest auscultation. IPF is the most common idiopathic fibrotic ILD, occurring more commonly in men older than 60 years of age [3]. Some patients present for evaluation of cough and dyspnea more than five years before being diagnosed with IPF, at times first receiving diagnoses of heart failure or chronic obstructive pulmonary disease [4]. Increasing clinician consideration of an ILD diagnosis in the appropriate context may shorten time from symptom onset to initial diagnosis in these cases (Table 1).

### 2.1. Initial Evaluation

When history and physical exam suggest the presence of a possible ILD, thorough clinical evaluation will include an environmental and drug exposure history, as well as hand, joint, and skin evaluation. Drugs most commonly associated with lung disease are cancer therapies (i.e., bleomycin, immune checkpoint inhibitors), rheumatologic agents, amiodarone, and antibiotics (i.e., nitrofurantoin) [16]. A thorough family history focusing on idiopathic interstitial pneumonia and autoimmune disease should also be performed [17,18]. Short telomere syndromes may be indicated by premature hair graying, hematologic abnormalities, and liver disease [19]. Although not generally utilized as a means of ILD diagnosis, testing of telomere length and telomere-related genes is available when there is concern for familial interstitial pneumonia [20].

Familiarity with common signs and symptoms of rheumatologic diseases including rheumatoid arthritis, systemic sclerosis, Sjogren’s syndrome, dermatomyositis, polymyositis, systemic lupus erythematosus, and antisynthetase syndrome will increase the level of suspicion for CTD-ILDs when these features are present. However, the pulmonary manifestations of rheumatologic illnesses in some cases may predate the development of other disease manifestations in the case of CTD-ILD; in particular, the inflammatory myopathies are known for this [11]. If there is a high index of suspicion for a CTD-ILD, the patient should also be referred for additional evaluation by a rheumatologist. Serologic testing commonly includes antinuclear antibodies, rheumatoid factor, cyclic citrullinated peptide antibodies, anti-neutrophil cytoplasmic antibody, creatine kinase, aldolase, ribonucleoprotein and antibodies against tRNA synthetases (Jo-1, PL-7, PL-12, etc.), Scl-70, anti-centromere, anti-RNA polymerase III antibodies, anti-Ro, anti-La, and double-stranded DNA in select cases [21].

If instead there is clinical concern for hypersensitivity pneumonitis, as in cases of known exposure to mold or birds in the home or workplace or based on the high-resolution computed tomography (HRCT) pattern, it is possible to evaluate levels of circulating immunoglobulins against various avian and microbial antigens in what is termed a hypersensitivity pneumonitis panel. However, many inciting antigens are not commercially available for testing, and many individuals with exposure but without HP test positive for serum IgG [7,22]. In practice, a large number of patients with biopsy-confirmed HP not only do not test positive for IgG, but never have a causative inhalational exposure identified [23,24]. In patients that do have an identifiable antigen exposure, an inhalation challenge involving the delivery of a nebulized antigen followed by physiologic monitoring has been described, though is not widely available in most centers [25,26,27,28]. Identification of the underlying exposure is beneficial when possible to allow for environmental remediation [29].

Pulmonary function testing should also be performed including spirometry, lung volumes, and the diffusion capacity for carbon monoxide (DLCO). Patients with ILDs typically exhibit reduced forced vital capacity (FVC), reduced total lung volume, and reduced diffusing capacities, though these values may appear normal early in the disease course, and in particular when combined pulmonary fibrosis and emphysema is present. Serial pulmonary function testing is generally performed to monitor for worsening physiology as a sign of disease progression once an ILD diagnosis has been established.

Recently there has been interest in characterizing pulmonary fibrosis following coronavirus disease 2019 (COVID-19) [30,31]. Pulmonary fibrosis is a known complication after acute respiratory distress syndrome (ARDS). Fortunately, in long-term follow up, patients with fibrotic changes following ARDS or infection with a separate coronavirus in 2003 generally demonstrated stability over time [32,33]. Studies specifically evaluating the long-term fibrotic effects of COVID-19 are ongoing, though early evidence suggests that the majority of these patients will not suffer from a progressive fibrosing process [34,35].

### 2.2. Screening

The relatives of patients with pulmonary fibrosis are at increased risk themselves of developing ILD [36]. Several studies have noted the presence of interstitial abnormalities on HRCT in asymptomatic relatives of pulmonary fibrosis patients, but the use of repeat HRCT as a screening mechanism in these individuals is probably not advisable based on radiation exposure and cost unless they are experiencing symptoms [37,38]. However, data regarding the use of alternate screening methods such as clinical evaluation and pulmonary function testing to reliably detect disease are limited and research is ongoing [39].

In terms of genetic predisposition, telomere-related genes and surfactant-related genes are most commonly associated with familial pulmonary fibrosis. It has been suggested that the sequencing of telomere and surfactant related genes be offered to any patient with fibrotic ILD who has at least one first or second degree relative with fibrotic ILD [5]. Telomere gene sequencing or telomere length testing can also be offered to any patient with suspected short telomere syndrome or an idiopathic fibrosing ILD that develops before the age of 50 [5]. First degree relatives of patients with short telomere syndrome can also be evaluated with a complete blood count and hepatic enzyme measurement, as these syndromes often manifest with bone marrow failure and liver disease [5].

The question of screening for ILD also arises in patients with known diagnosis of rheumatologic disorders. Given the significant morbidity and mortality associated with ILD in this patient population, early recognition and monitoring is essential and might improve with standardization of screening approaches in this setting [6].

### 2.3. High-Resolution Computed Tomography

When the level of suspicion is sufficiently high, HRCT of the lungs should be performed to determine whether imaging abnormalities are present and if so, to identify radiologic patterns which would aid in diagnosis. The usual interstitial pneumonia (UIP) pattern on HRCT is the hallmark of IPF, though is also observed frequently in cases of rheumatoid arthritis with ILD (RA-ILD) among other disease entities [40]. A joint collaboration between the American Thoracic Society, European Respiratory Society, Japanese Respiratory Society, and the Latin American Thoracic Society published updated guidelines in 2018 advocating the use of four diagnostic categories including “UIP pattern”, “probable UIP pattern”, “indeterminate for UIP”, and “alternative diagnosis” [41]. The presence of honeycombing is a distinguishing feature of the UIP pattern, which is typically subpleural with basilar predominance, though at times it may prove difficult to differentiate from paraseptal emphysema or traction bronchiolectasis [42]. The positive predictive value of radiographic UIP pattern for histopathologic UIP on biopsy has been reported as close to 90% [10,41,43,44]. When honeycombing is not present to designate radiographic UIP, a HRCT pattern of subpleural, basilar predominant reticular abnormalities with peripheral traction bronchiectasis or bronchiolectasis is termed probable UIP, and is also highly likely to have a histopathologic pattern of UIP [41,45].

Imaging may also identify a pattern of nonspecific interstitial pneumonia (NSIP), which can occur not only as an idiopathic entity but in a variety of conditions including CTD-ILD and drug-induced lung disease [12,46,47,48]. It is the most common radiographic pattern in cases of systemic sclerosis with ILD (SSc-ILD) [8]. NSIP is characterized by the presence of bilateral ground glass and reticular opacities that are usually lower lung-zone predominant [9].

Rather than UIP or NSIP patterns, imaging may demonstrate alternate findings that suggest various ILD diagnoses (Table 1) [48]. HP, for example, is radiographically suggested by centrilobular nodules and mosaic air trapping in an upper lobe distribution [13,14]. Sarcoidosis in contrast is characterized by hilar and mediastinal lymphadenopathy and bilateral nodules with lymphangitic spread, and architectural distortion when fibrotic [49,50].

### 2.4. Multidisciplinary Discussion

The process of accurately diagnosing a specific ILD is complex, and the gold standard for diagnosis involves the integration of clinical, radiographic, and possibly pathological information by a multidisciplinary team [41,51,52]. A multidisciplinary approach has been demonstrated to improve interobserver agreement and confidence level when diagnosing ILDs, and may even lead to a change in the initial diagnosis [53,54,55,56,57]. A recent Delphi survey and patient focus-group analysis identified the multidisciplinary conference as an essential component of an ILD clinic [58]. Multidisciplinary discussion (MDD) can be particularly important when HRCT findings are nonspecific, or there is discrepancy between the identified radiographic and histopathologic patterns [59]. A final diagnosis in certain patients may necessitate ongoing case review at interval multidisciplinary team meetings to re-evaluate updated clinical information and HRCT evolution, as disease behavior may further elucidate the diagnosis [59]. In addition to diagnosis, multidisciplinary team discussions are also commonly used to discuss patient management strategies [60].

### 2.5. Tissue Sampling

In cases where imaging and clinical findings are not diagnostic, more invasive methods of diagnosis may be pursued when deemed to be appropriate by the multidisciplinary care team [15,51,61]. Bronchoscopy with bronchoalveolar lavage, for example, may offer additional data pointing towards diagnoses such as chronic HP [62]. Endobronchial ultrasound-guided transbronchial needle aspiration, endobronchial, and transbronchial biopsy are commonly performed when sarcoidosis is suspected [63]. When tissue sampling is recommended to evaluate for UIP, surgical lung biopsy via video-assisted thoracoscopic surgery (VATS) is traditionally performed, ideally in at least two separate areas of the lung as there may be histologic differences between the sites [64]. The more common complications after these procedures include pneumothorax and postoperative pneumonia [65]. There are greater odds of postoperative mortality in patients who are older, male, with more comorbidities, on long term oxygen therapy, and undergoing open thoracotomy as opposed to VATS [65,66].

In recent years, there has been a growing interest in the use of transbronchial lung cryobiopsy (TBLC) for histopathologic ILD diagnosis [15,67]. This is in contrast to transbronchial forceps biopsy (TBB), which has previously been recommended against when IPF is a possibility due to low diagnostic yield [41,68]. As transbronchial sampling more readily targets the centrilobular portions of the lung, TBB can be reasonable to pursue for the evaluation of airway-centered disease processes such as HP and sarcoidosis. In contrast, establishing a diagnosis of UIP via transbronchial sampling even with transbronchial lung cryobiopsy (TBLC) is challenging due to the subpleural predominance of pathologic findings. Additionally, as a criterion for histologic UIP requires the absence of findings which would suggest an alternate diagnosis, sampling error with TBLC may lead to the unintentional exclusion of these features [69]. As such, TBLC is more likely to yield a diagnosis of probable UIP rather than definite UIP pattern compared to surgical lung biopsy [70].

Despite these limitations, COLDICE, a prospective comparative study in which patients underwent sequential TBLC and surgical lung biopsy, demonstrated high levels of agreement for MDD diagnosis [70]. Systematic reviews have demonstrated diagnostic yields of TBLC and SLB to be 80 percent and 90 percent, respectively, in cases of ILD of undetermined type [41,71]. Based on these findings, in addition to the consideration of TBLC being less invasive and less costly, recent guidelines suggest that TBLC is an acceptable alternative to SLB in medical centers with adequate experience in its performance when IPF or fibrotic HP are suspected [69,72].

As previously mentioned, in many instances biopsy is not felt to be necessary to establish a patient diagnosis following MDD [15]. For example, patients with HRCT patterns of definite or probable UIP generally do not require histologic confirmation unless there is clinical concern for a diagnosis other than IPF [73]. Similarly, a biopsy is not needed in patients with known CTD and HRCT findings consistent with CTD-ILD. In contrast, there are also cases where biopsy would be helpful to establish a diagnosis, but is impractical to obtain due to patient comorbidities or patient refusal. In these cases, multidisciplinary team discussions are particularly crucial to establish the most probable “working diagnosis” based on integration of all the available information, including demographic data, disease behavior before and after treatment, bronchoalveolar lavage findings, etc. [74,75,76].

### 2.6. Genomic Classifier

Recently a genomic classifier was developed in which lung tissue obtained via surgical or transbronchial biopsy undergoes whole transcriptome RNA sequencing. Subsequent gene expression analysis developed via machine learning distinguishes UIP from non-UIP histopathology [77,78]. A systematic review including four studies determined genomic classifier testing to identify a UIP pattern with a sensitivity and specificity of 68% and 92%, respectively [79]. The termed Envisia classifier has received regulatory approval for clinical use, although the most recent guidelines make no recommendation for or against its use [69].

### 2.7. Progressive Pulmonary Fibrosis

Once a fibrotic ILD has been identified, ongoing disease monitoring may reveal a progressive fibrosing process [3,80,81]. The term progressive pulmonary fibrosis (PPF) was recently coined in a joint clinical practice guideline to describe ILDs with radiologic fibrosis of known or unknown etiology excluding IPF who meet two of three criteria for disease progression. These criteria include: worsening respiratory symptoms, physiologic evidence of disease progression on pulmonary function testing (absolute decline in forced vital capacity ≥5% or absolute decline in diffusing capacity for carbon monoxide ≥10% predicted within one year of follow-up), and radiologic evidence of disease progression [69]. Radiologic disease progression can be manifested by new or increased traction bronchiectasis, ground glass opacities, reticular abnormalities, or honeycombing. The likelihood of a given non-IPF ILD exhibiting PPF is challenging to determine, but many ILDs are capable of this clinical phenotype, including idiopathic nonspecific interstitial pneumonia (NSIP), autoimmune ILDs, chronic hypersensitivity pneumonitis (HP), and chronic sarcoidosis among others [3,80,81].

Multiple definitions of disease progression have been used in clinical studies for selection criteria and measurement of treatment efficacy in the past, and this proposed PPF definition could serve to standardize research in the field. However, the grouping of various diseases into this progressive phenotype may risk oversight of the significant heterogeneity that exists under the PPF umbrella term. Further investigation of genetic factors or other biomarkers may have allowed for more robust phenotypic characterization [82]. One element of the PPF definition specifies worsening of disease over the course of a one year period; this does not allow for differentiation of patients who meet progressive criteria at the 6 month or at the 2 year mark, for example [83].

## 3. Pharmacologic Management

Treatment of ILDs is an active area of investigation, and in general is dependent upon the disease entity itself. In certain cases, treatment is encompassed by removal of the offending agent, as in drug-induced ILD, HP, or smoking-related ILDs. Additional non pharmacologic management strategies include environmental remediation in cases of HP, pulmonary rehabilitation, lung transplantation, and supplemental oxygen support among others, though description of these lies outside the scope of this review, which instead focuses on pharmacologic treatments.

### 3.1. Traditional Immunosuppressive Agents

Immunosuppressive therapy is a mainstay of treatment in many cases of non-IPF ILD. Ground glass opacification on HRCT is generally considered to represent a higher amount of cellularity in inflammatory-driven diseases, which may be more responsive to immunosuppression compared to imaging that is characterized primarily by fibrotic changes. Corticosteroids are often used first line and may be transitioned to non-steroidal agents in an effort to avoid the side effects of long term steroid use [84,85]. Non-steroidal agents utilized in the treatment of ILD include cyclophosphamide, mycophenolate, azathioprine, rituximab, and methotrexate among others (Table 2). There are few randomized controlled trials (RCTs) studying the use of such agents for the purposes of ILD, and current management strategies are largely based on observational studies and case series.

The RCTs that have been performed in large part specifically evaluate treatment of SSc-ILD and sarcoidosis. The Scleroderma Lung Study (SLS) I, for example, was a seminal placebo-controlled trial which demonstrated a significant treatment effect of oral cyclophosphamide on changes in FVC, as well as improvement in dyspnea and skin thickening in patients with SSc-ILD over a period of one year [94]. The Fibrosing Alveolitis in Scleroderma Trial (FAST) was another placebo-controlled trial using intravenous cyclophosphamide and oral prednisolone followed by maintenance azathioprine, which demonstrated a trend towards treatment benefit based on differences in FVC, although the difference did not reach statistical significance [98].

In SLS II, 142 patients were randomized to receive 24 months of mycophenolate mofetil or 12 months of oral cyclophosphamide followed by 12 months of placebo. Both treatment groups exhibited improvement in FVC at 24 months, though mycophenolate was better tolerated [99]. Mycophenolate is now commonly used as maintenance therapy not only for SSc-ILD, but many CTD-ILDs and HP based on SLS II and other studies which showed improvement or stability in lung function over time, as well as acceptable medication tolerability [100,101]. Mycophenolate sodium is an alternate medication formulation with an enteric coating that is often prescribed for patients who do not tolerate mycophenolate mofetil due to gastrointestinal side effects, though mycophenolate sodium has not formally been evaluated in controlled studies [85]. Azathioprine is often used in clinical contexts similar to mycophenolate mofetil based on the limited data that are available [101,102,103,104,105].

When treatment for pulmonary sarcoidosis is needed, the initial therapy for patients with symptomatic stage II and III disease is most often oral glucocorticoids as supported by several RCTs and the Cochrane review [106,107,108,109]. Stage IV disease may also be treated with glucocorticoids when a significant amount of ground glass is present on imaging and infection has been excluded, though most studies do not evaluate outcomes for patients with higher risk disease [110]. Because of the potential for spontaneous improvement in patients diagnosed with sarcoidosis, one group of investigators followed 149 patients from time of diagnosis for six months; during that time, 33 patients required glucocorticoid treatment due to clinical deterioration. After the six month observation period, 58 patients were noted to have had spontaneous disease improvement, while the remaining 58 patients had persistent stage II disease or higher. Patients with persistent disease were randomized to receive 18 months of glucocorticoid treatment at that time, or treatment with glucocorticoids only in case of functional decline. Of the patients in the latter group, 6 of the 31 patients required symptomatic treatment. On follow up, the investigators reported greater improvement in lung function, symptoms, and imaging for patients with disease that persisted for 6 months who received glucocorticoids compared to those patients treated only for symptoms [111].

In sarcoidosis patients who are treated with glucocorticoids and continue to experience disease progression, are unable to taper prednisone below 10 milligrams per day, or have unacceptable side effects from glucocorticoid therapy, methotrexate and azathioprine are common second-line agents [63,84,112]. One RCT randomized 138 patients with sarcoidosis to receive the anti-tumor necrosis factor agent infliximab or placebo and demonstrated increase in FVC from baseline to week 24 in the treatment group [113].

### 3.2. Methotrexate

Use of methotrexate is not uncommon in the treatment of patients specifically with RA or sarcoidosis. Concern regarding its capacity to cause pulmonary fibrosis has led to its discontinuation in some patients over the years. However, the incidence of methotrexate-induced lung disease may well have been overstated by the misperception that undiagnosed RA-ILD was caused by methotrexate use [114]. Recent studies published in 2020 have re-evaluated the link between methotrexate and ILD [115,116,117,118]. In the study by Juge et al., methotrexate exposure was examined in patients with RA-ILD and in patients with RA but without ILD [115]. The findings demonstrated that past methotrexate exposure was associated with a lower prevalence of RA-ILD, and that in patients with RA-ILD, methotrexate use was associated with longer time to diagnosis of ILD. All together these findings suggest that chronic fibrotic lung disease may not be an adverse outcome of methotrexate use, and that in the future methotrexate may instead be considered a reasonable treatment option for RA-ILD [114].

### 3.3. Biologic Agents

The use of rituximab, an anti-CD20 monoclonal antibody, is oftentimes reserved for patients with severe progressive disease when it is being prescribed for a primary ILD indication [85]. It has been demonstrated to stabilize or improve lung function and/or HRCT findings across a range of CTD-ILDs largely in retrospective and observational studies, with several RCTs currently ongoing [85,95,96,119,120,121]. One very recent RCT directly compared the use of rituximab versus cyclophosphamide in the treatment of CTD-ILD, which demonstrated similar efficacy overall and fewer adverse events in the rituximab group [97].

There is also interest in the use of abatacept and tocilizumab, an IL-6 receptor antagonist, for management of CTD-ILDs. A randomized controlled phase II trial (FaSScinate) of tocilizumab in SSC did not meet the primary endpoint of reduction in skin thickening, but did report evidence of less decline in FVC in the tocilizumab group [93]. A subsequent placebo-controlled phase III trial (FocuSSced) of tocilizumab in patients with SSc again did not meet the primary skin fibrosis endpoint, but treatment with tocilizumab was associated with significant attenuation in decline in lung function based on change in percentage of predicted FVC at 48 weeks [86]. While change in FVC was a secondary end point, and patients with SSc-ILD were not selectively enrolled, nonetheless the drug was FDA-approved for SSc-ILD [114].

### 3.4. Anti-Fibrotic Agents

The treatment of IPF has been marked by major revisions within the past ten years. Historically, patients with IPF were treated with immunosuppressive therapy until the PANTHER-IPF trial, which demonstrated overt harm in patients treated with prednisone, azathioprine, and N-acetylcysteine (10% mortality rate at interim analysis in the three-drug treatment arm compared to 1% mortality rate in the triple placebo arm) [90]. Two agents, nintedanib and pirfenidone, were approved by the FDA in 2014 to slow disease progression in IPF. Two RCTs (INPULSIS-1 and INPULSIS-2) compared nintedanib to placebo in patients with IPF and met the primary end point of reduced rate of annual decline in FVC compared to placebo [89]. Concurrently published were the results of the phase III RCT of pirfenidone versus placebo (ASCEND) in patients with IPF, which also demonstrated a reduced rate of FVC decline at 52 weeks in the treatment group [88]. Subsequently, the phase II randomized trial INJOURNEY investigated the combination therapy of nintedanib plus pirfenidone versus nintedanib monotherapy. The results indicated a manageable safety and tolerability profile in the combination therapy arm, supporting the need for further investigation into the efficacy of combination therapy in the future, although none has been reported to date [122].

Anti-fibrotic therapy has more recently been studied and used in the treatment of non-IPF fibrotic ILDs based on the hypothesis that there are likely to be similar pro-fibrotic disease pathways in IPF and non-IPF ILDs. The phase III RCT INBUILD assigned patients with non-IPF fibrosing lung diseases who met the study-specific criteria for ILD progression to receive nintedanib versus placebo. In the overall population, the annual rate of decline in FVC was significantly lower in the treatment group (−80.2 mL per year with nintedanib versus −187.8 mL per year with placebo, 95% confidence interval of the difference 65.4 to 148.5 mL; *p* < 0.001) [87]. The patients from the INBUILD study were later divided into five diagnostic subgroups (chronic HP, CTD-ILD, idiopathic NSIP, unclassifiable idiopathic interstitial pneumonia, and other ILDs), with analysis demonstrating consistent treatment effect across all five subgroups [123]. The SENSCIS RCT also demonstrated a smaller decline in FVC at 52 weeks with treatment of nintedanib compared to placebo specifically in patients with SSc-ILD [92].

Recently, a randomized placebo-controlled phase II trial evaluated the efficacy and safety of 24 weeks of pirfenidone treatment in patients with unclassifiable progressive fibrotic ILD. The study’s prespecified analysis was limited by intra-individual variability in home spirometry; however, the hospital-based spirometry demonstrated a reduction in decline in FVC in the treatment group [91]. Another phase II trial (TRAIL1) evaluated the efficacy of pirfenidone versus placebo specifically in RA-ILD patients; while the proportion of patients who met the composite primary endpoint including decline in FVC from baseline of 10% or more was not significantly different between the two groups, patients in the pirfenidone group had a slower annual rate of decline in absolute FVC and FVC percent [124]. The results of SLS III were also recently presented; this study evaluated combination therapy with mycophenolate mofetil and pirfenidone compared to mycophenolate and placebo in patients with SSc-ILD [125]. Results suggested that upfront combination therapy demonstrated more rapid improvement in FVC percent, as well as greater improvement in HRCT quantification of fibrosis and patient-reported outcomes [125]. Additional trials evaluating the utility of anti-fibrotic agents for non-IPF indications are ongoing [85].

### 3.5. Anti-Acid Treatments

In the past, antacid medications including proton pump inhibitors and/or histamine-2 receptor antagonists were recommended for patients with IPF in the hopes of improving respiratory outcomes in these patients [126]. This recommendation was based on observations that many IPF patients have gastroesophageal reflux (GER) and hiatal hernias, theoretical concerns over disease worsening due to micro-aspiration, and observational studies of survival benefit and reduction in FVC decline in treated patients [69,127,128]. However, a recent systematic review of fifteen studies that evaluated antacid medication use in patients with IPF found no statistically significant effect of treatment on disease progression, mortality, hospitalization rates, or disease exacerbations [129]. Of note, none of the reviewed studies specifically analyzed patients who were stratified as either having or not having confirmed GER, even though these medications may have a differential effect in these IPF subgroups. In the absence of definitive treatment benefits, most recent guidelines recommend against treating IPF patients with antacid medication in order to improve respiratory outcomes [69]. Randomized trials evaluating the use of antacid medication to compare respiratory outcomes in patients with IPF and GER were identified as a research need in the field [69]. In contrast, laparoscopic anti-reflux surgery in patients with IPF and abnormal acid GER is safe and well tolerated [130].

### 3.6. Pulmonary Hypertension Medications

Pulmonary hypertension due to interstitial lung disease (PH-ILD) is a known complication of ILD, with a prevalence of 30% to 50% in patients with advanced IPF, and is associated with significant morbidity and reduced survival [131,132]. Several clinical trials of pulmonary vasodilator therapy for this indication have not demonstrated treatment benefit, with some even demonstrating potential for harm [133]. However, most trials enrolled patients that were diagnosed with PH-ILD based on diffusing capacity and/or transthoracic echocardiogram rather than via right heart catheterization, and no trials have specifically evaluated treatment in severe PH-ILD likely due to the short life expectancy in this population [133]. In an exciting recent randomized, double-blinded, placebo-controlled trial of inhaled treprostinil in PH-ILD (INCREASE), at week 16 the treatment group demonstrated improvement in exercise capacity from baseline [134].

Of note, a diagnosis of World Health Organization (WHO) group 3 pulmonary hypertension is complicated in patients with underlying connective tissue disease, which themselves are well known causes of WHO group 1 pulmonary arterial hypertension. Additionally, pulmonary hypertension associated with sarcoidosis is included in WHO group 5, and is considered separately from other causes of fibrotic ILD.

Importantly, RCTs have also been conducted evaluating the use of pulmonary vasoactive drugs in ILD patients irrespective of pulmonary hypertension status. For example, the Sildenafil Trial of Exercise Performance in Idiopathic Pulmonary Fibrosis (STEP-IPF) investigated the use of sildenafil in patients with advanced IPF defined as carbon monoxide-diffusing capacity of less than 35% of the predicted value [135]. It was a negative study based on the primary endpoint of a 20% increase in 6-min walk distance, though did signal some efficacy in terms of arterial oxygenation, carbon monoxide diffusing capacity, degree of dyspnea, and quality of life. Similarly, the INSTAGE study evaluated the efficacy of co-administration of nintedanib and sildenafil in IPF patients with low carbon monoxide-diffusing capacities [136]. This was also a negative study with a primary endpoint of change from baseline in the total score on the St. George’s Respiratory Questionnaire; however, certain secondary endpoints trended towards benefit with the addition of sildenafil, such as rate of decline in FVC.

## 4. Conclusions

The timely diagnosis of ILD by a multidisciplinary team of providers is necessary to guide appropriate therapy and prognostic considerations. Recent advances in diagnosis include the use of TBLC and a genomic classifier to identify UIP following lung biopsy. Pharmacologic management of ILD has advanced over the years, perhaps most notably to include the use of anti-fibrotic medications to slow disease progression in IPF. The results from the recent INBUILD study have supported the extension of anti-fibrotic drug use in progressive non-IPF fibrotic ILDs as well, perhaps hinting at a common fibrotic pathway that bridges disease entities. Recently the INCREASE study demonstrated the benefit of inhaled treprostinil in PH-ILD, one of the few positive studies for treatment of this disease sequela. Future studies will hopefully continue to improve our understanding of these complicated disease processes, and test novel agents to improve outcomes for patients with these diseases. Providers should encourage appropriate patients to enroll in observational or interventional trials to facilitate the achievement of these goals.

## Figures and Tables

**Table 1:** Notable clinical, radiologic, and histologic features of common fibrotic interstitial lung disease subtypes and their management.

Interstitial Lung Disease	Clinical Features	Imaging	Histology	Management
Idiopathic Pulmonary Fibrosis [1,5,6]	Male predominance, age > 60 years, finger clubbing, bibasilar crackles, restrictive pattern on pulmonary function tests	Definite or probable UIP pattern; peripheral and basal predominant, reticulation, bronchiectasis, honeycombing	Spatially and temporally heterogeneous fibrosis with subpleural predominance, architectural distortion, fibroblastic foci	Antifibrotic therapy, referral for lung transplant evaluation
Hypersensitivity Pneumonitis [5,7,8,9]	Inspiratory squeaks, prolonged exposure to inhaled antigens (commonly mold and avian), may have positive hypersensitivity panel, lymphocytosis on bronchoalveolar lavage	Upper lobe predominant, centrilobular nodules, peribroncho-vascular distribution, ground glass opacification, mosaic attenuation, expiratory air trapping, three density pattern (formerly head cheese sign)	Peribronchiolar interstitial pneumonia, airway-centered fibrosis, poorly formed non-necrotizing granulomas	Antigen avoidance, environmental remediation, glucocorticoids or immunosuppressive therapy, antifibrotic therapy for progressive fibrotic disease
Idiopathic Nonspecific Interstitial Pneumonia (NSIP) [10]	Female predominance, crackles on pulmonary auscultation, restrictive pattern on pulmonary function tests	Peripheral and basilar predominance though can be diffusely distributed, ground glass opacities, symmetric, immediate subpleural sparing, traction bronchiectasis in fibrotic disease	Interstitial thickening with abnormal alveolar septa, temporal homogeneity, honeycombing possible	Immunosuppressive therapy, antifibrotic therapy for progressive fibrotic disease
Systemic Sclerosis Interstitial Lung Disease [11,12]	Skin thickening, Raynaud’s phenomenon, telangiectasias, GER, fingertip lesions, concomitant pulmonary hypertension, abnormal nailfold capillaroscopy, positive antibodies including anti-Scl-70, anticentromere, or anti-RNA polymerase III	NSIP pattern more commonly than UIP pattern	NSIP pattern most commonly	Immunosuppressive therapy, antifibrotic therapy for progressive fibrotic disease
Rheumatoid Arthritis Interstitial Lung Disease [5,11]	Erosive arthritis, synovitis, morning stiffness, rheumatoid nodules, anti-cyclic citrullinated peptide (CCP) antibodies, rheumatoid factor (RF) less specific	UIP pattern more commonly than NSIP pattern	Both UIP and NSIP pattern, lymphoid hyperplasia	Immunosuppressive therapy, antifibrotic therapy for progressive fibrotic disease
Sarcoidosis [13,14,15]	Multisystem organ involvement most commonly including skin, eyes, heart, liver, and lymphatics; lymphocytosis on bronchoalveolar lavage	Early stages characterized by bilateral hilar lymphadenopathy and/or pulmonary infiltrates, perilymphatic micronodules; Scadding Stage IV disease characterized by fibrosis with architectural distortion or bullae	Well-formed non-necrotizing granulomas with possible coalescence, giant cells	Monitoring in non-extensive, asymptomatic disease followed by first line glucocorticoid therapy and alternate immunosuppressive agents if needed

**Table 2:** Common pharmacologic therapeutics for interstitial lung diseases.

Medication	Mechanism of Action	Monitoring Parameters	Adverse Effects	Highlighted Trials
Nintedanib	Tyrosine kinase inhibitor	Hepatic function	Diarrhea, nausea/vomiting, elevated transaminases, arterial thromboembolic events have been reported	INPULSIS [86] SENSCIS [87] INBUILD [88] INJOURNEY [89]
Pirfenidone	Pleiotropic effects including regulation of transforming growth factor beta and tumor necrosis factor alpha	Hepatic function, weight	Photosensitivity, gastrointestinal intolerance, elevated transaminases	ASCEND [90] SLS III [91] INJOURNEY [89] TRAIL1 [92]
Methotrexate	Folate antimetabolite	Complete blood count, renal function, hepatic function; pregnancy test, hepatitis screen, and interferon-gamma release assay prior to initiation	Pneumonitis, diarrhea, hepatotoxicity, alopecia, photosensitivity, bone marrow suppression	
Corticosteroids	Interference in leukocyte function	Blood pressure, serum glucose, weight, bone mineral density, electrolytes, hemoglobin	Including cataract formation, increased intraocular pressure, hyperglycemia, skin changes, fluid retention, peptic ulcer, psychologic disturbance, decreased bone density, adrenal insufficiency	PANTHER [93]
Mycophenolic Acid	Inhibits B and T cell proliferation	Complete blood count, blood pressure	Hypertension, edema, skin rash, leukopenia, nausea/vomiting, constipation, decline in renal function, gastric ulcers, low risk lymphoma	SLS I and II [84,94]
Azathioprine	Inhibits purine synthesis	Complete blood count, renal function, hepatic function; thiopurine S-methyltransferase testing prior to initiation	Leukopenia, hepatotoxicity, gastrointestinal intolerance, opportunistic infection	PANTHER [93]
Cyclophosphamide	Alkylating agent	Complete blood count, renal function, urinalysis	Bone marrow suppression, alopecia, hemorrhagic cystitis, bladder cancer, cardiotoxicity, opportunistic infection	SLS I and II [84,94] FAST [85]
Rituximab	Monoclonal antibody targeting CD20-postive B lymphocytes	Complete blood count, hepatitis panel, and interferon-gamma release assay pre-infusion	Infusion reaction, acute pneumonitis, opportunistic infection	RECITAL [95]
Tocilizumab	Monoclonal antibody to inhibit interleukin-6	Complete blood count, hepatic function, and interferon-gamma release assay pre-infusion	Opportunistic infections including fungal and tuberculosis	FaSScinate [96] FocuSSced [97]

## Data Availability

No new data were created or analyzed in this study. Data sharing is not applicable to this article.
